# Household Gods

**Published:** 1875-07

**Authors:** 


HOUSEHOLD GODS—The permanent objects with which a man surrounds himself and his family, are certain to exert a powerful influence upon their taste and culture. You cannot furnish your son or your daughter with cheap looking illy-constructed furniture, without impairing his or her taste and lowering the mental and moral tone. Somebody affirms that a badly-shaped sofa, a rickety table or a broken chair, is sufficient to demoralize any observing person in one year.

We think there is much truth in the old adage the "the best is the cheapest." In nothing is this statement more nearly correct than in the matter of household furniture. In our climate, solidity is the one thing needful, and when this is combined with tasteful forms, there is little more to be asked. In the furniture of Messrs De Graffe and Cochrane. No. 152 west 23d street, these two essentials appear to be fully complied with. The styles into which the solid wood is cut, are symmetrical and pleasing while the woods used are so carefully seasoned and the several parts are so securely united, that the goods are able to withstand the dampness, and cold and heat, of our trying climate, without shrinking, or straining, or cracking. The prices, moreover, seem to be fair and reasonable, as low probably, as can be afforded for such work; as low, we are assured, as many dealers place upon work of a weak and inferior kind. It will surely be useful for some of our readers to lay up in the store-house of memory, a fact which we have never heard disputed, that articles of furniture bought at this large establishment, are at once moderate in price, substantial in construction and elegant in design.

---

SCIENCE IN STORY.—Do our friends reflect that we are giving them a most valuable manual of the household in the serial which we are publishing from month to month, entitled "Our Summer at Maplewood"? We believe it to be the most valuable addition to domestic literature which has ever appeared in print. One lady tells us that the chapter on bread-making is worth to her more than the $25 which she has paid in subscriptions to our magazine for herself and as presents to friends. We supply the back numbers at reguar rates.

---

Beware of copious drinks of ice-water when you are over heated.

